# Offender Recovery. Forensic Patient Perspectives on Long-Term Personal Recovery Processes

**DOI:** 10.3390/ijerph18126260

**Published:** 2021-06-09

**Authors:** Jette Møllerhøj

**Affiliations:** Competence Centre for Forensic Psychiatry, Mental Health Centre Sct. Hans, DK-4000 Roskilde, Denmark; jette.moellerhoej@regionh.dk

**Keywords:** mentally disordered offenders, recovery processes, offender recovery, stigma, roles of professionals, values, approaches

## Abstract

Knowledge on user experiences from mentally disordered offenders (MDOs) is still limited in a Danish context, especially regarding recovery from offences, severe mental illness, long-term admissions and often involuntarily contact with hospital psychiatry. The study is based on 34 semi-structured interviews with nine forensic patients exploring their experiences with personal recovery processes. The MDOs point out a significant number of elements and factors enhancing, supporting and limiting personal recovery processes. Long-term recovery processes for MDOs involve coming to terms with mental disorders as well as offences. Working with offender recovery implies addressing and understanding the index offence leading to psychiatric measurement as well as addressing risk and prevention of future crime. This coming to terms is an individual and deeply personal process and it often involves several and changing narratives. According to the informants, professionals play a crucial role in supporting recovery processes and maintaining hope and optimism over time. MDOs experience structural barriers limiting recovery potential, especially stigma or limited areas of participation. It is important not to focus solely on personal recovery as a one-dimensional individual process or responsibility, but as a process also marked by structural and organisational challenges.

## 1. Introduction

Over the past 10 years, political focus on personal recovery, participation, involvement, empowerment and user perspectives has increased. As a consequence, principles of recovery are central in today’s mental health services, and patients are supposed to receive recovery-oriented care and treatment, focusing on hope, possibilities and empowerment in their lives. This focus is reflected in a number of Danish policy papers as well as activities and projects such as user-controlled admissions, employment of peer workers, recovery schools, implementation of Shared Decision Making, and focus on the patients’ goals for care and treatment, as well as patients taking part in the development of their treatment plans [1,2,3,4,5].

The strategic aim of recovery orientation also includes patients under restraint according to the Danish Mental Health Act as well as mentally disordered offenders with placement or treatment orders. However, although strategy papers refer to the concept of personal recovery, recovery still seems to be a slippery concept with a number of meanings in clinical practice. For instance, personal recovery seems to be confused with the concept of recovery as clinical remission [6]. Furthermore, mental health professionals often express bewilderment and perplexity regarding how to support individual recovery processes and how to act in practice as coaches rather than experts [7,8].

Some of this insecurity as to how to react as a health professional and support recovery-oriented care might be reduced by increased knowledge on what patients consider as important and helpful ingredients in their recovery processes. The international literature on personal recovery has grown intensively, and there is also a substantial amount of literature on personal recovery processes and forensic psychiatry [7,9,10,11,12,13,14,15,16]. There is a widespread consensus in the literature on the importance of focusing on and supporting recovery for patients struggling with mental disorder as well as offences, eventually even further complicated by substance use issues. Likewise, it is generally agreed that crucial recovery elements to a large extent are the same for forensic patients as well as non-forensic patients [12]. At the same time, however, it is important to consider the complexity within the forensic patient population as well as special and judicial circumstances framing trajectories for mentally disordered offenders, because these formal or structural circumstances may delay or complicate rehabilitation and recovery processes [12,13,17].

However, knowledge on user experiences from MDOs is still limited in a Danish context, especially regarding recovery from offences, severe mental illness, long-term admissions and often involuntarily contact with hospital psychiatry [17,18,19,20]. Over the past 20 years there has been a remarkable growth in the number of individuals sentenced to either placement or treatment within the hospital psychiatry in Denmark, from a prevalence of 1445 persons in 2001 to a prevalence of app. 3–4000 today. The most complex MDOs are treated in specialised forensic services. The vast majority of MDOs are treated as outpatients or admitted in general psychiatric units [21]. Consequently, they are in contact with a number of professionals in various settings during their often long term sentences. Since the aim of the sentence to either placement or treatment is to prevent recidivism to future offences, it seems crucial if and how professionals can enhance and support the individual’s way towards a satisfying and hopeful life within the law. However, our knowledge on how to support and reach the overall goal of preventing future crime is rather limited [21,22,23].

The aim of this paper is to provide new and in-depth knowledge of forensic patient perspectives on elements enhancing, supporting and limiting personal recovery processes.

## 2. Materials and Methods

The study is based on semi-structured interviews with forensic patients exploring their experiences with personal recovery processes. This was part of a larger qualitative interview study focusing on turning points before and after the informants’ debuts as mentally disordered offenders and sentences to either treatment or placement [22].

The study was conducted within the mental health services in the Capital Region of Denmark. In total 38 interviews with 23 informants from 10 different clinical pathways were carried out. Both patients and caregivers were interviewed, as well as a few professionals and one victim. This paper is focused on patient perspectives and is based on 34 interviews with nine patients.

When asking patients about key elements in their recovery processes, the point of departure in this study is taken from Anthony’s concept of personal recovery as “a deeply personal, unique process of changing one’s attitudes, values, feelings, goals, skills and/or roles. It is a way of living a satisfying, hopeful, and contributing life even within the limitations caused by illness” [24]. Furthermore, as pointed out by, for instance, Alain Topor, one has to be aware of the social interaction between an individual and the social context and not solely reduce a recovery process to an individual and personal matter or responsibility [25].

The study is also inspired by the method of biographical narratives [26,27] and the importance of the personal narrative and identity-shaping as part of recovery processes [28].

### 2.1. Setting

The mental health services in the Capital Region are the largest of their kind in a Danish context and provide care for approximately one third of the total number of psychiatric patients in Denmark [29]. They also provide care for a large number of mentally disordered offenders; of whom some are treated in specialised forensic services as inpatients or followed in forensic assertive community treatment teams. However, the vast majority of MDOs are outpatients and are cared for in general psychiatric teams and units [21].

### 2.2. Participants and Recruitment

Informants were recruited thanks to health professionals informing patients about the study. Patients consented to be contacted by the research unit, and agreements about interviews were made.

The patients in this study represent various experiences from general psychiatric as well as specialised forensic services. They recount experiences from various pathways and phases, i.e., before, during and after a sentence to treatment or placement. Six of them have had 10 years or more contact with mental health services. Five out of nine had not committed previous crime. Their offences, which led to the actual sentence to either placement or treatment in a psychiatric institution, were all violent crimes; varying from robbery, threats, kidnapping, rape and attempted manslaughter to actual manslaughter. The patients are 23–40 years old, seven of the participants are male and two are female. The majority are ethnic Danes. They all lived from public benefits by the time of the interviews, but more than half of them have previous working experience. Their household situation varies (from owner-occupied dwellings to homelessness) as does their educational level (no elementary school to unfinished university degree). None of them is in a relationship, but one is a parent of minor children.

In total the informants are experienced and represent many aspects of pathways, circumstances and ways into forensic psychiatry. They contribute different as well as similar ways of dealing with this situation. The paper will argue that these experiences are relevant and transferable to other patients with forensic psychiatric pathways. Although forensic service users may be the predominant concept currently, the informants in this study are referred to as patients, due to their involuntary settlement with mental health services, which they cannot renounce. Consequently, it does not make sense to paraphrase them as service users.

### 2.3. Thematic Analysis

The semi-structured interviews were conducted around a thematic interview guide consisting of the following themes: current situation, turning points, current and previous contact with hospital psychiatry, current and previous contact with municipalities, criminal offences, childhood and education, working experience, everyday life and social relations, substance use, relations with professionals, the role as a patient, treatment aims, experiences with mental health issues, and the future.

The interviews were recorded, transcribed verbatim and analysed using content and textual analysis focusing on identifying patterns and themes [30,31]. The empirical material has been exposed to vertical as well as horizontal analysis in order to identify significant statements and phrases within each interview as well as across all interviews [32]. A number of in vivo citations are used throughout the analysis. All informants are referred to as he, in order to ensure anonymity.

## 3. Results

All together, the informants point towards a number of elements supporting and enhancing personal recovery processes:

- Variety in activities during hospitalisation as well as during outpatient pathways
- Something meaningful to do during the day
- Approach challenges in a positive way
- Take on responsibility for acts and one’s own life
- Get to know symptoms and mental condition and how to react
- Take one day at a time
- Accept help and support from professionals
- Accept help and support from network and relatives
- Ensure a safe place to live and a stable income

The informants in this study have gone through a large number of shifts during their pathways as forensic patients. They describe various phases, each with distinctive challenges. Help and support from their network and relatives have been crucial during all phases. Support from network and relatives consists of a number of actions, from taking care of practical chores and acting as case manager across welfare institutions and their logics to maintaining hope and representing persistent belief in improvement on behalf of the patient [22]. As one patient puts it: “It’s good when someone from your network or family can push you a little. Sometimes, what is needed is simply a kick in the ass”. According to another informant, the help from relatives is literarily what kept him holding on: “If my mum hadn’t been there, I wouldn’t have stayed alive”. Whilst the patients appreciate support from their network and relatives, they also try to protect them and shield them from certain knowledge or situations. “I didn’t want my mum to know about this”, one informant said, as “she has been through enough”. For instance, patients choose to protect their relatives from seeing episodes of mechanical restraint, or from being present in court listening to charges and details from police reports regarding the offence committed by the patient.

Interaction with professionals is another crucial theme according to the informants. Most of them point towards the importance of being approached in a friendly, respectful, open-minded and curious manner by professionals. One informant describes an important meeting with new members of staff: “They smiled. And suddenly, I felt like a human being again”. Another informant underlines critical professional actions during a hospitalisation: “It meant the world to me, that they [members of staff] were there, took care of me, paid attention and that they were visible in the environment [in the unit]”.

Moreover, informants list time and perseverance as vital elements facilitating recovery processes. Several of them have experienced how changes take place slowly and during long processes. Most of the informants have gone through long admissions in specialised forensic units with limitations and restrictions. Slowly, they have moved towards less intensive care and more freedom. One informant describes the remarkable step from being on short-term granted leave with members of staff to what is experienced as freedom: The capability to go fishing on one’s own away from the hospital area. This process has taken more than 10 years. When telling about it he underlines patience and perseverance as fundamental necessities: “You must keep on believing that things will change and get better”. For others the processes have been more abrupt; shifting between hospitalisation and outpatient services. For a large number of mentally disordered offenders in Denmark with sentences to treatment, this back and forth between admissions and outpatient treatment is the common pattern. They are hospitalised when in risk of recidivism to crime due to relapse, substance use or social circumstances, and they are being treated in outpatient services when their condition is stable [23].

Time is likewise an important factor in the long-term work of getting to know symptoms and early signs of relapse. As one informant describes, it has been a long intensive job to get to know various aspects of the mental disorder and what to do “when my brain plays tricks on me”. It has been a “slow haul” and it is still ongoing: “I try to work with myself. That’s the only thing one can do”.

Last, but not least, antipsychotic medication is also described as a pivot in several of the pathways in this study. Attitudes to and experiences from psychopharmacological treatment are varied and diverse amongst a psychiatric patient population. In this study, the informants represent the widespread experience that medication has been and continues to be an important and helpful element. Several of them have previously experienced medication non-adherence or insufficient medical therapy, and this played a crucial role in the situations that led to criminal offences. Medication then becomes an important issue in the future prevention of recidivism to crime. The informants have during their forensic pathways participated in decisions regarding choice or dose of medication, and this may be one explanation as to why they consider and accept steady medicine as an important fulcrum in their recovery processes. However, it is considered one amongst several central elements supporting and enhancing recovery processes.

### 3.1. Offender Recovery

It is well-described in previous studies on recovery in general psychiatric settings how coming to terms with severe mental illness can be a long and demanding journey. For mentally disordered offenders, this task is even more complex; in addition, they also need to come to terms with the offences committed [10,11].

The patients in this study have spent much time figuring out how to frame and reframe their pathway and how they ended up with a sentence to treatment or placement. For some of them, it has been a challenge to establish a narrative about this; i.e., a narrative that works for themselves or a narrative that works convincingly towards family, friends or their network. These narratives are not necessarily identical, and they can also change over time. Most of the informants have had to work on several adjusted explanations and sustainable narratives.

For several, it was a challenge to find out how to tell close family members about the serious offences committed. Often the patients worry about the reactions from relatives, and whether the relatives will be able to accommodate the offences and keep in contact with the patient. One patient told his relatives about the offence in a letter. He has not talked to them about the content of the letter, but he emphasises that the relatives have declared themselves willing to come and visit while he is admitted: “They say we just have to get through this, and what has happened has happened. Now we have to look ahead”.

### 3.2. Mental Illness as Explanatory Frame

For some of the informants, the mental disorder becomes a crucial factor in understanding and explaining the offences. Consequently, the offences committed occurred because of the outburst or relapse of mental illness, as one informant puts it: “Normally, I’m the kind of person who would never hurt a fly, but I was psychotic [at the time of the crime] and I couldn’t help it”. Referring to mental illness, and some sort of connection between illness and offence, underlines the offence as an undeserved act. This explanatory model or narrative is useful to some of the informants, and it has helped them come to terms with the in many ways incomprehensible sequence of events leading to severe offences towards innocent victims.

However, a reference to illness does not work for all informants. Some of them do not feel ill, or at least not so ill that it qualifies for the vague term “psychotic”, neither now nor at the time of the crime. In these cases, the comprehension of offences as consequences of severe mental illness is not a sustainable narrative. For some of the informants who have taken on illness as an explanatory figure, this leads to new speculations and worries: “If I did this because of illness, does this mean that it could happen again, if I fall ill again?” Another patient experienced that using the illness to explain why he ended up with a sentence to treatment led to new questions and assumptions among his friends. For instance, they focused on him being a dangerous person with a mental disorder rather than seeing him as a friend being honest with them about previous difficulties.

### 3.3. Guilt and Shame

The Danish penal law is not based on M’Naghten rules and does not apply the not guilty by reason of insanity principle. Instead the mentally disordered offender is regarded as legally guilty, but not punishable.

Personal guilt is a central theme to most of the informants. Two of the informants are convicted for manslaughter. In both cases the victims were close relatives. The pathways for these two patients are marked by tremendous guilt, and at the same time they grieve the loss of a family member. The double sorrow for one’s own action and for one’s own loss has previously been described in prison populations as a heavy and complex burden [33,34]. However, there is also a risk that the offender’s own grief is neglected or not seen as legitimate. For one of the patients, it took several months before he realised that the victim was his own mother: “For a long time I thought it was a robot and it was my right [to fight]. So, it really hurts to find out that it was my mother”. According to this informant, he has benefited from sessions with a psychologist. Furthermore, it has been helpful to watch one of the recent national television shows about patients from another forensic specialised unit: “I saw another boy, like me, also suffering from schizophrenia, who had also killed his mum. He said that it was his illness that got his mum killed. I recognise this as I would never have hurt my mum [under normal circumstances]”.

In cases such as this, close family members have to deal with their own loss as well as whether and how to keep and maintain contact with the patient, who has caused tremendous sorrow. For the patient, the type of reactions and support from relatives is crucial: “I’m so relieved that they haven’t cut me off, because they realised how ill I was [at the time of the offence]”. Together this family has established the narrative that the mother “sacrificed her life, so that he [the son] could get better”.

Apart from the two informants convicted for manslaughter, the general picture in this study are offences towards unknown and in many cases arbitrary victims. For a number of informants, it has taken up a great deal of time to realise that their actions have harmed other people while they were in the wrong place at the wrong time, or on duty as professionals. Recognising this often leads to intense feelings of guilt: “I didn’t know them. They were just there shopping. I got upset when I realised this”. Another patient explains how he faces how much damage has been done to the health professionals involved in the incident: “When hearing about it [in court] I realised how much damage it had done to them and how frightened they must have been. This is horrible”.

Along with guilt, shame is also predominant among the patients interviewed. Furthermore, several informants feel an urge to explain themselves and the circumstances, as well as to excuse themselves to the victims. One of the patients has written a letter to the victims of the threatening offence trying to explain that he did not intend to harm them: “It had nothing to do with them personally and I am not a monster harming others”.

Another patient had eye contact with a victim in the courtroom. No words were exchanged, but the patient experienced a strong mutual understanding and remittance as “she smiled towards me”. In this pathway we also interviewed the victim, and she spontaneously told about the same moment in court: “We had a reasonable eye contact and exchanged smiles at last. Like, sort of: Okay, here we are, shit happens, but we have to get on from here”. The victim also experienced this encounter as a de-escalating and mitigating turning point. However, there do not seem to be any systematic practices or scheme supporting mediation and conciliation between victims and mentally disordered offenders. Several of the informants express that they have missed settings and opportunities to come to terms with the often severe offences committed, and that they lack confident and significant relations where this coming to terms could take place.

Guilt and shame can also be related to mental disease or life circumstances [35,36], and not necessarily solely to criminal offences. Either way, this complex reflects an enormous amount of feelings and thoughts to come to terms with as a mentally disordered offender. It is often said that mentally disordered offenders suffer from anosognesia or completely deny the offences leading to psychiatric measures. This is not the case for the informants in this study. However, it is important to bear in mind that their comprehension of their symptoms and actions vary and may have changed a number of times during their clinical pathways.

### 3.4. Long-Term Processing

For several patients, it has been and still is a long-term process to establish sustainable explanatory models and narratives about illness and offences. Some of them were offered the “due to underserved illness” frame as defendants in custody before trial. For some, this recognition of the previous situation as chaotic came along with the increasing effect of anti-psychotic medication after a couple of months. Several describe how they at the time of the offence saw and experienced things which they later on realise were closely related to psychosis or delusions. 

Some have found it helpful when professionals have suggested a specific frame or narrative to encompass the serious events, whereas others to a larger degree have relied on relatives in the process of dealing with the situation and its implications. Their perspectives and experiences vary between the informants, and to a large degree this is related to where in a clinical pathway they are situated by the time of the interviews. For some, the offences which led to their sentence to treatment took place recently, and the need to come to terms with them is more present. For others, several years have passed, and their offences are not as present, or they have spent a great amount of energy over the years to come to terms with the whole situation as mentally disordered offenders. Either way, they all represent the experience that it can be a long and demanding process coming to terms with and establishing meaningful narratives about aspects of illness, offences and their whole situation with long-term psychiatric measures.

Most of the informants have developed a number of narratives to work in different settings. For instance, one narrative is used when telling close family or friends about the situation as a mentally disordered offender, whereas a less detailed narrative is used towards network or distant family members. Furthermore, the narratives are adjusted over time as circumstances change or things are seen in a new perspective. Some of the informants have chosen silence as a strategy and do not talk about the offences or the shift from being a patient in general psychiatry to becoming a forensic patient. To remain silent about certain aspects also proves to be a demanding job, and it often involves worries as to whether family or network will find out about the offence in the past.

Most of the informants report the need for someone to talk to and someone who can support this long-term processing of events and circumstances leading to their path as mentally disordered offenders. Most of them also describe a lack of confident interlocutors over time. “No, we haven’t talked much”, one informant says about the relation to the contact person in the mental health services. “I have received a sentence to treatment”, another informant says, “but it hasn’t changed much in my treatment and treatment plan”.

### 3.5. Limiting Factors and Obstacles to Recovery Processes

The informants have also experienced a number of factors counteracting with recovery processes.

For most of the mentally disordered offenders, the sentence to placement or treatment implies long-term and involuntary dealings with mental health services. The experience of being forced to be in contact with professionals and services not chosen by oneself can be an obstacle to a fulfilling life characterised by a large degree of empowerment and autonomy. For some of the participants, it has taken a while coming to terms with the psychiatric measure as a structural condition which one has to deal with one way or the other, as it is sanctioned by the court.

Other structural issues such as the experience of differential treatment, or the experiences of stigma are at stake for several of the informants. Internal stigma in interaction with health professionals and health services as well as external stigma in society is at stake for a number of patients, not least mentally disordered offenders [37]. Furthermore, representations in the media of mentally disordered as monstrous and dangerous to a large degree affect the patients as such representations may influence how network and relatives perceive the individual patient and his acts and illness. “So I think they’ll distance themselves from me, because they think of me as dangerous and ill”, one informant says, whereas another has experienced that “two of my friends could not deal with me becoming mentally disordered, and they thought I was too dangerous to be with due to the mental illness”. Several of the informants have spent a lot of time worrying about how members of their network will react and whether they will be capable of accommodating mental health issues and offences. This illustrates how experienced stigma can be transferred and internalised into self-stigma. It is well documented how negative representations, pessimism and stereotyping affect hope and belief in possible changes. Furthermore, stigma and self-stigma can reduce the ability to formulate new narratives and self-perceptions beyond offences and mental disorders. From a sentence to treatment being lifted, it takes five years for the previously mentally disordered offender to obtain a clean criminal record. For one of the informants in the study, this has been tremendously demanding to deal with in the years after cessation of measures: “I feel labelled and branded and I’m still ashamed over my criminal record and mental illness”.

Experiences of injustice or unequal access to support can also be an obstacle in a recovery process. Some of the informants have experienced their trial as a show trial, where everything was already decided, and where they did not feel justice in legal proceedings and the sanction meted out by the court. Some of them did not feel that they had sufficient legal aid and support as their defense lawyer did not have specific experience or knowledge of forensic psychiatric matters. Some informants focus on lack of proportion and believe that if they had been found punishable and sentenced to prison, they would have served the sentence and then been over and out within a certain period of time. Instead, they are left with uncertainty as to how long the sentence to treatment is supposed to go on. Some have experienced up to one year in custody before the trial. If sentenced to placement or treatment, the previous time spent in custody is not taken into consideration, whereas it would be deducted in a sanction to prison. Some of the informants consider this deeply unfair. It supports their feeling of being deprived of influence on their own life and trajectory.

For some of the informants, challenges regarding access to substance use treatment have been a barrier, as well as the absence of supported housing or issues related to means of subsistence. The absence of social support has played a major role in the potential recovery processes and a critical role as a risk factor in relation to the offences which led to the psychiatric measures in several of the pathways studied.

Interaction and sustainable and perseverant relations with friends, network and professionals are according to the informants, crucial elements supporting and enhancing recovery processes. Consequently, the opposite can obstruct or limit potential recovery. Absence of a sustainable therapeutic relation can be detrimental in relation to recovery processes. Some of the informants have experienced encounters with mental health professionals where they were not met in respectful, open-minded or acknowledging ways. “I’ve had many unpleasant experiences with members of staff. I felt I was treated like an animal”, one informant says. Others have experienced absent or distanced members of staff hard to reach and get into contact with when needed: “The staff spent a lot of time in the staff room, looking at their mobile phones, chatting and doing lots of things. I think they were busy with a lot of things, except taking care of the patients”.

Some of the informants have experienced that they were expected to act or behave in certain ways in order to obtain help or support: “If you want to be part of this mental health business, you as a patient have to fit into a specific box. And if you’re not willing to submit, then mental health services cannot help”, one informant says. Another has experienced that he should express specific symptoms in certain ways to get attention and to be seen as someone having a relevant and worthy need of help: “One has to talk to the inner voices constantly or appear psychotic, shout at the staff or throw furniture in the living room in order to get in contact with members of staff”. According to this informant, he appeared too quiet and properly dressed, and consequently his need for help and support was not accommodated.

## 4. Discussion

The central factors supporting recovery as pointed towards by the informants in this study are in line with previous studies on recovery in forensic contexts [10,11,12,13,14,15,19,20,21,22,23,24,25,26,27,28,29,30,31,32,33,34,35,36,37,38].

The elements and factors pointed out by the informants also correspond with the CHIME model maintaining:

- Connectedness
- Hope and optimism
- Identity
- Meaning of life
- Empowerment

The elements in CHIME are crucial in recovery processes and have been experienced and described by a large number of service users [9]. The quality of the relation between patient and significant others, not least professionals, has also been identified as a crux in personal recovery processes [16,39,40,41]. The professionals play an important role as carriers of hope and optimism over time, which enhances the patient’s opportunities to create new narratives and identities. This underlines what has also previously been suggested: Maybe it is not so much about specific interventions or nursing actions, but much more about how care is delivered and what approach and view of humanity are at stake [16,42,43]. What matters most to the mentally disordered offender is to be met in an accommodating, open-minded, curious and respectful way.

A compassionate and non-judgmental approach seems pivotal in order to address the index offence which led to the sentence to placement or treatment in cooperation with the patient. A therapeutic approach to a patient’s offence demands dedicated and stage-specific interventions from the mental health professional as well as courage and willingness to reflect on one’s own prior understanding and emotional reactions to severe offences [44]. The process of offender recovery is twofold: to address the index crime and circumstances which led to it, as well as addressing and reducing the risk of re-offending [45]. Working with offender recovery seems crucial in order to support identity-building and new understandings and meanings of life, and to overcome consequences of severe mental illness and committing crimes [10]. Some of the informants addressed limited access or reduced possibilities to participate in society due to stigma or structural obstacles towards obtaining a clean criminal record. This underlines the importance of connectedness in recovery processes, and this seems to be hard for some MDOs to achieve. Are recovery processes for MDOs somewhat different from personal recovery processes for general psychiatric patients, one might ask? In order to reduce stigma, it seems prudent to address the number of similarities between recovery processes and what elements supporting and enhancing them, irrespective of the legal status of the patients. At the same time, however, one must not turn the blind eye to the complexity (psychopathology/co-morbidity/social marginalisation) of the forensic patient population and the double task of care and control always framing recovery processes for MDOs; taking care of the mentally ill offender while at the same time protecting the surrounding society and/or victims of the patient’s offence. 

As part of the offender recovery process, this study has identified a strong need amongst the informants to explain themselves and apologise to the victims. However, no systematic frame to facilitate this exists in a psychiatric context. It is relevant to consider whether experiences with negotiating conflicts and mediation in relation to criminal offences dealt with in the prison and probation services are transferable to a psychiatric setting [46,47]. Furthermore, several of the informants suffer from guilt and shame, and express needs to process this. Until now, processes of dealing with sorrow, guilt and shame in relation to severe offences have only been described in prison populations [33,34]. Future research on this subject in a psychiatric context is highly relevant.

### 4.1. Dilemmas, Paradoxes, Pitfalls

It has been shown in a number of papers that recovery is also possible when it comes to forensic patients, and that it is possible to find the delicate balance between increased self-determination and participation for the individual patient, while at the same time taking into account that this rehabilitation and recovery process has to be safe for the surrounding community [7,17].

However, there is still a risk that what we are working on is some sort of pseudo-involvement or pseudo-participation, especially in relation to MDOs with long-term hospitalisation. Inpatient treatment as well as a sentence to placement or treatment notoriously means limiting autonomy empowerment and the possibilities of taking charge of one’s own life. One way of dealing with this dilemma or risk of romanticising a forensic recovery process is to point towards the time span, the legal frame and the double task regarding the patient as well as the surrounding society and the victims. Recovery processes in a forensic context are possible, but they often involve a very long time span, and a huge number of back and forths, small and large turning points, relapses and obstacles.

The informants in this study describe a number of factors and elements in recovery processes. Some of them belong in the health care sector and are related to health care legislation, but most of them relate to social legislation and social welfare. Patients are, and should be, concerned with their needs and not with division of labour or organisational logic, or lack thereof. However, it is well-described how patients with complex needs and problems are at risk of falling through the cracks. Danish mental health care is organised and divided between five regions running hospital psychiatry and outpatient services, and 98 municipalities of various sizes running and funding psychosocial rehabilitation (social psychiatry). This poses a number of challenges as to consistent courses of action and cross-sectoral co-operation that may support the individual recovery process. This underlines the importance of structural and organisational factors and how they can influence, and in the worst case limit, recovery potential. Consequently, we should be cautious and not focus only on personal recovery as a one-dimensional individual process or responsibility, but as a process with social interaction and context [9].

The informants in this study experienced that they had to navigate in certain ways in order to be worthy and legitimate to receive help and support. Such experiences have also been found previously amongst general psychiatric patients and clients seeking help in the social security system [48,49]. This calls for further studies on what expectations MDOs face in their encounter with mental health services or social services. Furthermore, it also calls for reflection on what happens to patients not capable of navigating and honoring these expectations. Finally, we need to consider how to deal with patients who refuse to work on their recovery processes in line with what is expected in the welfare state context. What if they do not want to recover? What if they do not want increased power or saying in their own lives, but prefer to go down or go a completely different way [7,9]?

### 4.2. Strengths and Limitations of the Study

The chosen study design using individual in-depth semi-structured interviews enables a unique access to personal experiences. The nine informants represent a variety in pathways and life circumstances. Several interviews/points of impact with most of the informants have helped towards a deeper understanding of challenges and experiences over time as well as insight in the various stages and phases of a pathway structured by a sentence to placement or treatment. 

All informants have in various ways and to certain extents come to terms with suffering from severe mental disorders as well as having committed offences. Furthermore, most of them view and accept psychopharmaceutical treatment as a necessary tool in their recovery processes. This does not fit the psychiatric patient population in general, not least the group of mentally disordered offenders. MDOs are often in clinical practice represented as suffering from anosognosia and/or denying the offences as charged [9]. Consequently, it could be argued, the informants in this study represent the compliant or the well-behaved. However, most of the informants have also previously been in some sort of denial, and their current understanding of the situation at the time of the interviews has come along over time. The study design could perhaps have benefited from recruiting informants with completely different views on illness and offences, but then they would not have been sources to shed light on various phases in recovery processes, only sources to a certain early phase. All in all, the various and large number of experiences expressed by this study population are believed to be transferable and will be recognised and shared by other mentally disordered offenders.

## 5. Conclusions

When asked, MDOs point out a large number of elements and factors supporting, enhancing or limiting their personal recovery processes. Long-term recovery processes for mentally disordered offenders involve coming to terms with mental disorders as well as offences. Working with offender recovery implies addressing and understanding the index offence leading to psychiatric measurement as well as addressing risk and prevention of future crime. This coming to terms is an individual and deeply personal process and it often involves several and changing narratives over time.

According to the informants, the professionals play a crucial role in supporting recovery processes and maintaining hope and optimism. The approach, values and perseverance at stake seem more important to patients than specific professional acts or interventions. However, several of the informants describe a lack of confident and significant relations to support their coming to terms over time.

MDOs experience structural barriers limiting recovery potential, not least stigma, generalisations or monstrous narratives about forensic patients, or limited access or possibilities to participate in the community. It is important not to focus solely on personal recovery as a one-dimensional individual process or responsibility, but as a process with social interaction, context, structural and organisational challenges.

## Data Availability

Not applicable.
